# What Is the Right Carbon for Practical Anode in Alkali Metal Ion Batteries?

**DOI:** 10.1002/smsc.202000063

**Published:** 2021-02-02

**Authors:** Jun Zhang, Junwei Han, Qinbai Yun, Qi Li, Yu Long, Guowei Ling, Chen Zhang, Quan-Hong Yang

**Affiliations:** ^1^ Nanoyang Group State Key Laboratory of Chemical Engineering School of Chemical Engineering and Technology Tianjin University/Collaborative Innovation Center of Chemical Science and Engineering Tianjin 300350 China; ^2^ Joint School of National University of Singapore Tianjin University, International Campus of Tianjin University Binhai New City Fuzhou 350207 China; ^3^ Department of Chemistry City University of Hong Kong Hong Kong China; ^4^ School of Marine Science and Technology Tianjin University Tianjin 300072 China

**Keywords:** alkali metal ion batteries, alloy anodes, carbon anodes, carbon frameworks, conductive additives

## Abstract

Carbon materials have great potential for being the anode of choice in alkali metal ion batteries and are also crucial for constructing an efficient spatial framework for the production of alloy anodes with higher capacities. For the design of practical carbon anodes, the criteria of sufficient charge storage, a high initial coulombic efficiency, and excellent stability are proposed, which calls for the selection and optimization of the carbon microstructure as well as the matching of electrolytes. For the design of the carbon framework for alloy anodes, the principles of interfacial cohesion, spatial interconnection, and structural stability are proposed, thus recommending a proactive design strategy for better stability and volumetric performance. Research history together with representative research progress is reviewed and discussed in detail in an attempt to stimulate more research interest and promote ideas for the critical search for the right carbon to use as an anode in alkali metal ion batteries. Lastly, specific bottlenecks restricting the successful transfer of these carbons from laboratory to industry are highlighted. The importance of a precise understanding of the charge storage mechanism, the development of matching electrolytes, and the ability to produce the necessary carbon framework in large quantity for higher capacity alloy anodes are discussed.

## Graphical abstract

Carbon materials are potential for being the practical anodes in alkali metal ion batteries (AMIBs) and also crucial for constructing an efficient spatial framework for alloy anodes with higher capacities. Research history together with representative research progress is reviewed and discussed in an attempt to promote ideas regarding the critical search for the right carbon for practical anodes in AMIBs.

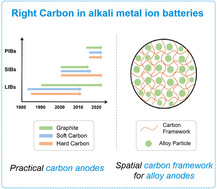

## 1 Introduction

Recent years have seen significant research and commercialization progress in the field of alkali metal ion batteries (AMIBs) other than lithium‐ion batteries (LIBs), to meet the increasing demands of portable electronics, electric vehicles, and large‐scale energy storage systems. As the most important component of the batteries, the selection of electrode materials determines some critical parameters such as the energy density, initial coulombic efficiency (ICE), and cycling stability. Therefore, researchers have spared no effort in developing practical electrodes for different applications.^[^ ^1^, ^2^, ^3^, ^4^, ^5^, ^6^, ^7^, ^8^, ^9^, ^10^, ^11^, ^12^, ^13^ ^]^

Carbon materials are the most practical candidates for anodes due to their low cost, natural abundance, easy production, high conductivity, adjustable microstructure, and surface chemistry.^[^ ^6^, ^14^, ^15^, ^16^, ^17^, ^18^, ^19^ ^]^ As the most representative example, the graphite anode has dramatically pushed forward the commercialization of LIBs.^[^ ^20^, ^21^, ^22^, ^23^ ^]^ The highly reversible formation of graphite intercalation compounds (GICs) and a specially modified interfacial electrochemistry are key to this.^[^ ^23^, ^24^, ^25^, ^26^, ^27^, ^28^, ^29^, ^30^, ^31^ ^]^ To meet the ever‐increasing demand for high‐energy‐density LIBs, durable and conductive carbon frameworks have been designed to support high‐capacity alloy anodes to construct a continuous conductive network and suppress the volume change.^[^ ^32^, ^33^, ^34^, ^35^, ^36^, ^37^, ^38^, ^39^, ^40^, ^41^, ^42^, ^43^, ^44^, ^45^, ^46^ ^]^ Both sodium‐ion batteries (SIBs) and potassium‐ion batteries (PIBs), the counterparts of LIBs, have developed rapidly in recent years, and carbon materials have also been actively investigated for use in them.^[^ ^47^, ^48^, ^49^, ^50^, ^51^, ^52^ ^]^ Similar carbon frameworks have also been designed and constructed for high‐capacity alloy anodes.^[^ ^53^, ^54^, ^55^ ^]^ Although design of the carbon has seen significant progress, there is still plenty of room for optimizing the nanostructures to produce a more energetic, faster charging, safer, and longer life battery. A logical review of previous studies and a guide for the future carbon structural designs is urgent and valuable to the field.

In this review, guidelines for designing practical carbon anodes and efficient carbon frameworks for alloy anodes are carefully proposed. Earlier and present research progress is then reviewed, together with a discussion of the most important contributions, in an attempt to provide a clear picture of what researchers have done and their significance. The importance of a precise understanding of the charge storage mechanism and the development of matching electrolytes for these anodes, as well as the ability to massively produce carbon–alloy composites with well‐controlled spatial framework are then considered. We hope this review will stimulate research interest in this field and thus promote the development of advanced carbon designs for AMIBs.

## 2 Guidelines for Carbon Designs

### 2.1 Practical Carbon Anodes

In principle, the energy density of a battery is calculated by the product of the reversible capacity and the open‐circuit voltage. Specifically, the reversible capacity is determined by the capacity of reversible charge transfer per unit weight (Ah g^−1^) between anode and cathode, and the open‐circuit voltage by the difference between the electrochemical potentials of the anode and cathode.^[^ ^56^ ^]^ Therefore, to increase the energy density when a certain cathode is selected, the electrochemical potential of the anode should be as low as possible (higher than 0 V) without sacrificing safety and stability. Moreover, the reversible capacity of the anode should be as high as possible without sacrificing reversibility. Ideally, a lithium metal anode is supposed to be the best option. However, its safety concerns have been the biggest obstacle in restricting its practical use since the 1980s, especially when coupled with liquid electrolytes. As a result, researchers have mostly turned to carbon materials because of their excellent electrochemical reactivity and diverse structures that allow one to tune the reactivity.^[^ ^5^, ^21^, ^57^, ^58^, ^59^, ^60^, ^61^, ^62^ ^]^

**Figure 1:**
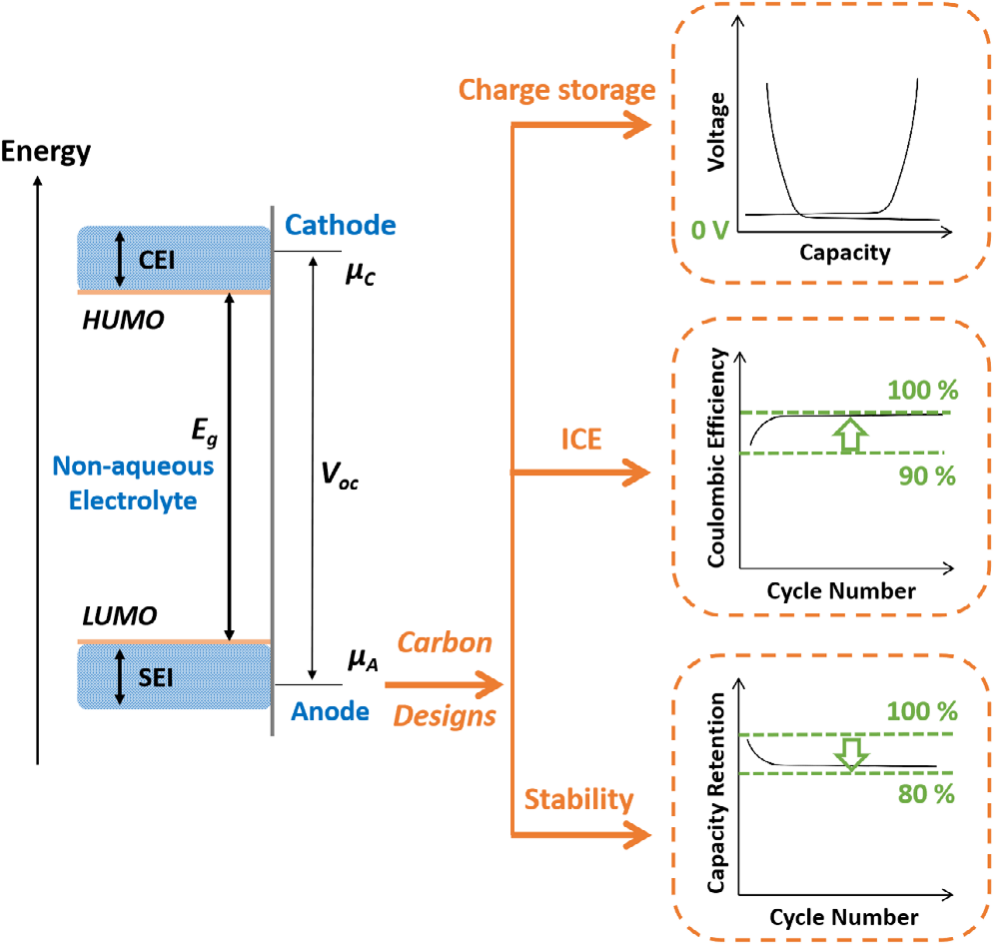
Illustration of the open‐circuit energy diagram and the most critical electrochemical parameters guiding the design of a carbon for the practical anode. *E*_g_ is the thermodynamic stability window of the electrolyte, namely, the difference between the HOMO and LUMO of the electrolyte. *μ*_A_ and *μ*_C_ are, respectively, the electrochemical potentials of the anode and cathode and *V*_oc_ is the open‐circuit voltage of the battery, which is equal to the difference between *μ*_C_ and *μ*_A_. To achieve effective carbon designs for anodes, sufficient charge storage, high ICE, and excellent stability are the basic electrochemical requirements for practical applications.

Only one specific type of carbon material has the potential to be the optimum anode and the reason lies in the charge storage mechanism. For example, activated carbon, the commercial electrode material for supercapacitors, has a large specific surface area (SSA) and stores energy through ion adsorption on the surface. However, it is not suitable for batteries due to the lack of sufficient charge storage, resulting in inferior energy output. Typically, a high energy barrier is needed to activate a battery‐like redox reaction which delivers a much higher energy output and has a voltage plateau at relatively low voltage. As shown in **Figure** **1**, sufficient charge storage in the carbon anode leads to the appearance of a low‐voltage plateau and this region should principally contribute to the total reversible capacity. Therefore, a precise understanding of the charge storage mechanisms of different carbon materials in different AMIB systems is beneficial for selecting the right carbon anode.

In addition, as shown in Figure 1, for nonaqueous electrolytes, their highest occupied molecular orbital (HOMO) and lowest unoccupied molecular orbital (LUMO) are theoretically calculated to be constants, which determine the thermodynamically stable voltage window.^[^ ^63^, ^64^ ^]^ However, for carbon anodes, their lower limit of operating voltage is far below this stable window, resulting in the massive decomposition of the electrolyte to form a solid electrolyte interphase (SEI) during the initial discharge. This passivating SEI is supposed to protect the carbon anodes from further decomposition. Ideally, the selected electrolytes should have a controlled minimum decomposition when matched with a specific carbon anode to increase the ICE.^[^ ^65^, ^66^ ^]^ As shown in Figure 1, the ICE should be higher than 90% and the coulombic efficiency of the following cycles should be close to 100%. In addition, the solvation and desolvation properties of various solvent molecules must be given careful consideration because they have a profound impact on the charge storage process of the carbon anode. Last but not the least, the long‐term stability influenced by the interfacial electrochemistry must also be optimized to meet high‐standard commercial requirements. As shown in Figure 1, after hundreds of cycles, the capacity retention should be higher than 80% if we define the capacity retention of initial cycle as 100%.

The layer structure of graphite is suitable for the intercalation of lithium ions to form highly reversible and stable GICs, resulting in a relatively long voltage plateau near 0.1 V versus Li/Li^+^. Specifically, low‐stage LiC_6_ is the major GIC after the electrochemical storage of lithium ions in graphite, theoretically delivering a specific capacity of 372 mA h g^−1^. Similarly, in PIBs the low‐stage KC_8_ is the major GIC with a theoretical capacity of 279 mA h g^−1^, whereas for SIBs the intercalation of sodium ions into graphite is thermodynamically difficult, and only high‐stage GICs can be formed such as NaC_64_. In addition, the successful commercialization of graphite in LIBs is closely related to the incorporation of ethylene carbonate (EC) into the carbonate‐based electrolyte, which produces a protective SEI to greatly improve the ICE and cycling stability. As a result of combining graphite with EC solvent, an ICE over 90%, a reversible capacity over 350 mA h g^−1^, and steady cycling over 500 cycles are obtained and basically meet industrial demands.

### 2.2 Efficient Carbon Frameworks for Alloy Anodes

Elements in group IV or V can form high‐capacity alloying compounds due to their reversible alloying reaction at relatively low potentials.^[^ ^45^, ^67^, ^68^, ^69^ ^]^ However, the alloying reaction usually leads to severe volume expansion and even continuous self‐pulverization of the electrode materials,^[^ ^70^, ^71^, ^72^ ^]^ which decreases the cycling stability and shortens the lifespan of the electrode. Taking a silicon anode in LIBs as an example, its highest reversible capacity makes it the most promising candidate for future high‐energy‐density LIBs, while a dramatic volume change of over 400% is also a big obstacle to conquer before practical use.^[^ ^68^, ^71^, ^73^, ^74^, ^75^ ^]^ Various solutions have been proposed such as nanostructuring, fabricating a multidimensional structure, and composite design.^[^ ^67^, ^76^, ^77^ ^]^ Among them, composite design is the most cost‐effective and achievable, especially when coupled with different carbons. From the viewpoints of both research and industry, alloy anodes coupled with carbons are the way to develop high‐capacity anodes for AMIBs.

**Figure 2:**
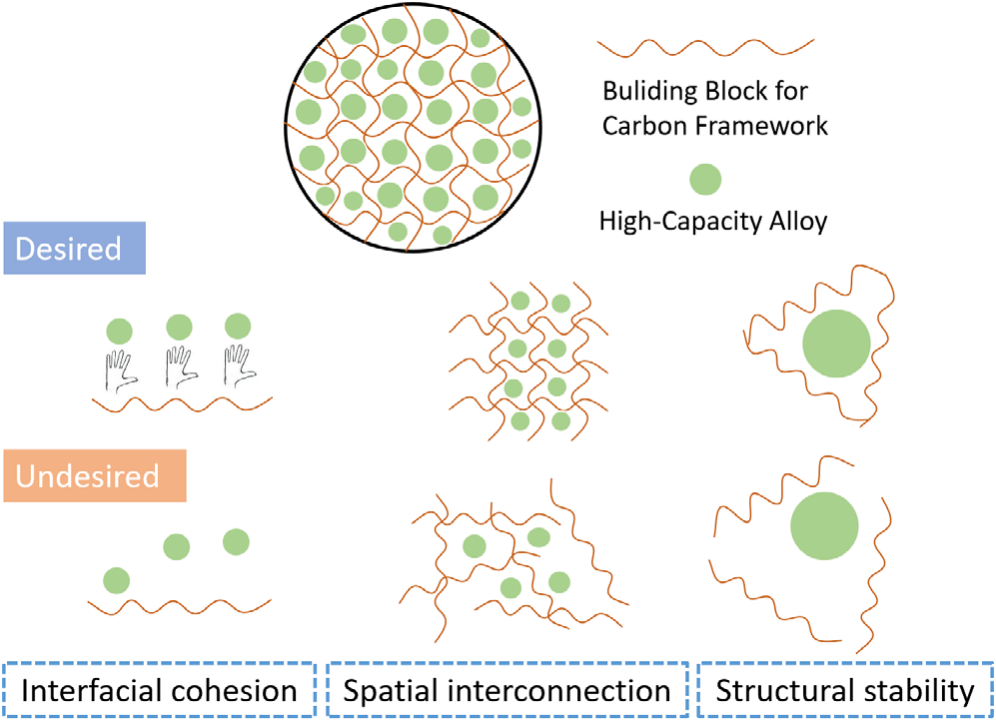
Schematic of the preferred properties of a carbon framework loaded with high‐capacity alloys. First, the desired basic building block for carbon framework should have specific electrostatic interaction or chemical bonding to guarantee the tight interfacial cohesion with alloy particles, while the undesired building block for carbon framework would lack necessary interfacial cohesion and lose contact with carbon framework during alloying reaction. Second, the desired carbon framework should have an excellent spatial interconnection which provides abundant available space for the incorporation of more alloy particles, while the undesired carbon framework lacks spatial interconnection and provides less available space for alloy particles. Third, the desired carbon framework should possess structural stability which keeps integrated during alloying reaction, while the undesired carbon framework would be broken due to the apparent volume change of alloy particles.

To construct efficient carbon frameworks, the features shown in **Figure** **2** have to be considered. The first desirable feature is interfacial cohesion. For the alloy particles dispersed in carbon framework, the large volume change during charge storage may potentially fracture the material and break the electrical continuity, leading to fast capacity fade. To effectively retain the electrical continuity, the interfacial cohesion between the alloy and the carbon should be strong, which usually means the presence of chemical bonding or electrostatic interaction. The second feature is spatial interconnection which will not only improve the electrical continuity but also increase the number of electrochemically active alloy particles inside the carbon framework, thus contributing to a higher volumetric capacity of the composite electrode. Moreover, for a stable and protective carbon framework with spatial interconnections, there is less possibility of the continuous formation of a SEI as a result of the exposure of cracked alloy particles to the electrolyte. The third feature is structural stability. With a stable and elastic multidimensional carbon framework, the quick relaxation of stress produced by unavoidable expansion and shrinkage of the alloy can be achieved.

## 3 Practical Carbon Anodes

**Figure 3:**
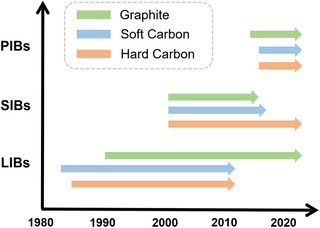
A brief research history of practical carbon anodes in AMIBs.

As already discussed in Section 2.1, there are some explicit principles for designing the right carbon anode for real applications. A brief summary of the research history of practical carbon anodes in AMIBs is shown in **Figure** **3**. The earliest investigation of carbon anodes in AMIBs was the use of soft carbon in LIBs around 1983. After that, hard carbon also attracted a lot of attention in LIBs. It was not until 1990 that graphite was shown to be irreplacable in LIBs. For SIBs, the search for the right carbon started around 2000. Unfortunately, graphite was soon shown to be unsuitable, while soft carbon was supposed to have potential for high‐power applications. Hard carbon is now the widely accepted anode for SIBs and related research is on‐going. For PIBs, graphite has been investigated since 2015 and promising electrochemical performance can be delivered. Both soft and hard carbon started to be investigated after 2015, with some promising electrochemical characteristics.

### 3.1 Carbon Anodes for LIBs

The 2019 Nobel Prize for Chemistry was awarded to three distinguished scientists who made rechargeable LIBs possible. Akira Yoshino, one of the laureates, is recognized as the first person to develop the commercially available LIBs and initially use carbon materials as the anode. Graphite is now the dominant option for the anode of commercial LIBs, but it fails to show an acceptable initial electrochemical performance because of its side reactions with electrolytes. Taking a polycarbonate (PC)‐based electrolyte for example, the cointercalation of PC and lithium ions into graphite is thermodynamically favorable, which leads to severe gassing issues and even exfoliation of the graphite. As a result, the expected low‐stage lithium GICs have seldom been synthesized electrochemically. Developing nongraphitic carbons and modifying the electrolytes are two fundamental solutions to this problem.

For nongraphitic carbons, researchers have mainly focused on soft and hard carbons to stabilize carbon anodes in PC‐based nonaqueous electrolytes. Nongraphitic carbons cannot be exfoliated by PC‐based electrolytes, but give a sloping charge/discharge curve ranging from 1.0 to 0.0 V versus Li/Li^+^. Nongraphitic carbons are apparently inferior to graphite regarding the energy output, but greatly improve the cycling stability and safety of LIBs. Therefore, the early‐generation LIBs used nongraphitic carbons as the anode in PC‐based electrolytes. For the first‐generation carbon anode, low‐cost petroleum coke was used, which is a typical soft carbon and available in large quantities from the petrochemical industry.^[^ ^78^, ^79^ ^]^ Unfortunately, this kind of soft carbon can only deliver half the capacity of graphite. Because of the belief that increasing the graphite interlayer spacing would facilitate the lithium storage and increase the specific capacity, the second‐generation carbon anode focused on hard carbons. Experimentally, hard carbon synthesized from poly(furfuryl alcohol) resin delivers a reversible capacity slightly higher than graphite. However, hard carbon anodes still have a sloping voltage profile and low ICE. The capacitive‐like lithium storage characteristics of both soft and hard carbons originate in the poorly defined stoichiometry of their lithiation compounds. Despite the improved reversibility of nongraphitic carbons, they are still not the ideal anode hosts from an energetics perspective.

**Figure 4:**
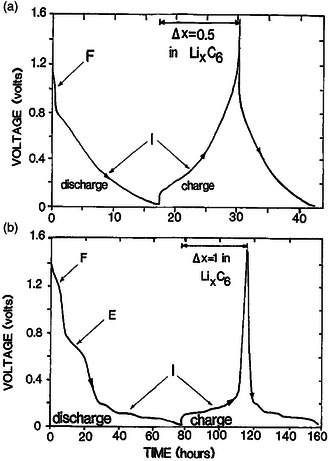
Comparison of the potential profiles of a) soft carbon (petroleum coke as the precursor) at cycle 2 and 99, and b) graphite at cycle 2 and 19. Reproduced with permission.^[^ ^81^ ^]^ Copyright 1990, IOP Publishing.

To modify the electrolyte, two commonly used nonaqueous electrolytes including carbonate‐based and ether‐based have attracted the most attention.^[^ ^80^ ^]^ Ether‐based electrolytes are intrinsically stable against electrochemical reduction; however, ether‐assisted formation of ternary GICs takes place at the high‐voltage plateau which decreases *V*_oc_ and induces an energy penalty. Therefore, the optimum solvent should be unstable against reduction to induce the preferred decomposition, and more importantly its decomposition should introduce a protective solid layer on the graphite surface to prohibit further decomposition as well as unexpected cointercalation of solvent molecules. This goal is ambitious, but EC is definitely a surprise. Combining EC with other carbonate solvents, the SEI mostly forms on the edges and surfaces of the graphite, which effectively restricts cointercalation and further electrolyte decomposition, thus greatly improving the cycling stability of the graphite anode. Moreover, the formation of such an SEI is highly efficient and the ICE is also improved, which is also critical for the practicality of the graphite anode. Dahn et al. initially introduced EC as a component into PC‐based electrolytes and explored the lithium intercalation characteristics of both soft carbon and graphite, as shown in **Figure** **4**.^[^ ^81^ ^]^ During the first discharge, irreversible reactions associated with the formation of a SEI on the surface of carbon were detected, and the reaction stops when the SEI fully covers the available carbon surface. With such a protective SEI, the graphite anode delivers excellent cycling stability in the following cycles.

After the combination of a highly graphitic carbon and an electrolyte that contains EC but not PC or ether‐based solvents, graphite is protected from both cointercalation and exfoliation and is able to be fully lithiated to LiC_6,_ which contributes to the higher energy density of LIBs when matched with a specific cathode. Moreover, graphite is readily obtained with a much lower cost, which makes it the commercialized anode material. Tarascon et al. reported that EC‐based electrolytes are stable against oxidation up to high potentials and are suitable for use with most high‐voltage transition metal oxide cathodes.^[^ ^82^ ^]^ As a result, a graphite anode together with an EC solvent have become indispensable in commercial LIBs ever since.

In the battery industry, there is a strict restriction on the SSA of graphite, namely, that it must be lower than 2 m^2^ g^−1^. The targeted modification of the microstructure and morphology of raw graphite is therefore essential before electrode preparation. For graphene with a much higher SSA, the interaction between its electrochemically active surface and the electrolyte is extremely intense, leading to a large irreversible capacity and an extremely low ICE. In addition, the charge storage mechanism of graphene is mostly based on the adsorption of lithium ions on the surface, leading to an inferior energy density. Therefore, graphene and other large‐surface‐area carbons are not appropriate for use in LIBs, but proved to be feasible as the anode in lithium‐ion capacitors (LICs). For both soft and hard carbons, which have improved reaction kinetics, better cycling stability and safety than graphite, they also have attracted a lot of attention from researchers and been actively investigated for high‐power applications in LIBs.

### 3.2 Carbon Anodes for SIBs

Sodium is the alkali metal counterpart to lithium and shares similar physical and chemical properties. Due to the abundance and lower cost of sodium over lithium, SIBs are better for large‐scale stationary energy storage applications. Learning from the experience of LIBs, graphite was initially investigated as the anode for SIBs; however, the electrochemical intercalation of sodium ions into graphite is rather limited.^[^ ^83^, ^84^ ^]^ There are mainly two approaches to promote the use of graphite in SIBs, the first is using highly reversible cointercalation of sodium ions and ether solvent into graphite.^[^ ^85^, ^86^, ^87^ ^]^ However, the average redox potential is higher than 0.5 V versus Na/Na^+^, while the reversible capacity is lower than 150 mA h g^−1^. Another is using partially reduced graphite oxide to increase the interlayer spacing.^[^ ^88^ ^]^ Unfortunately, the low‐voltage plateau related to the formation of GIC is missing and only a capacitive‐like sloping voltage profile is obtained.

Soft carbons also give a sloping voltage characteristic similar to graphite. The optimum soft carbons show reversible capacities in the range of 200–250 mA h g^−1^ and an average working potential near 0.5 V v ersu s Na/Na^+^. They have recently been reported to demonstrate excellent rate capability, indicating their potential for use in high‐power SIBs. Hard carbon has been tested and shown to deliver a reversible capacity higher than 300 mA h g^−1^ with a large part of this coming from the low‐voltage plateau below 0.1 V versus Na/Na^+^. As a result it is widely accepted as the most practical anode for SIBs.^[^ ^89^ ^]^ During recent years researchers have been dedicated to developing high‐performance hard carbon anodes and high reversible capacities ranging from 250 to 350 mA h g^−1^ have been obtained with a relatively low average working potential of 0.2 V versus Na/Na^+^.

**Figure 5:**
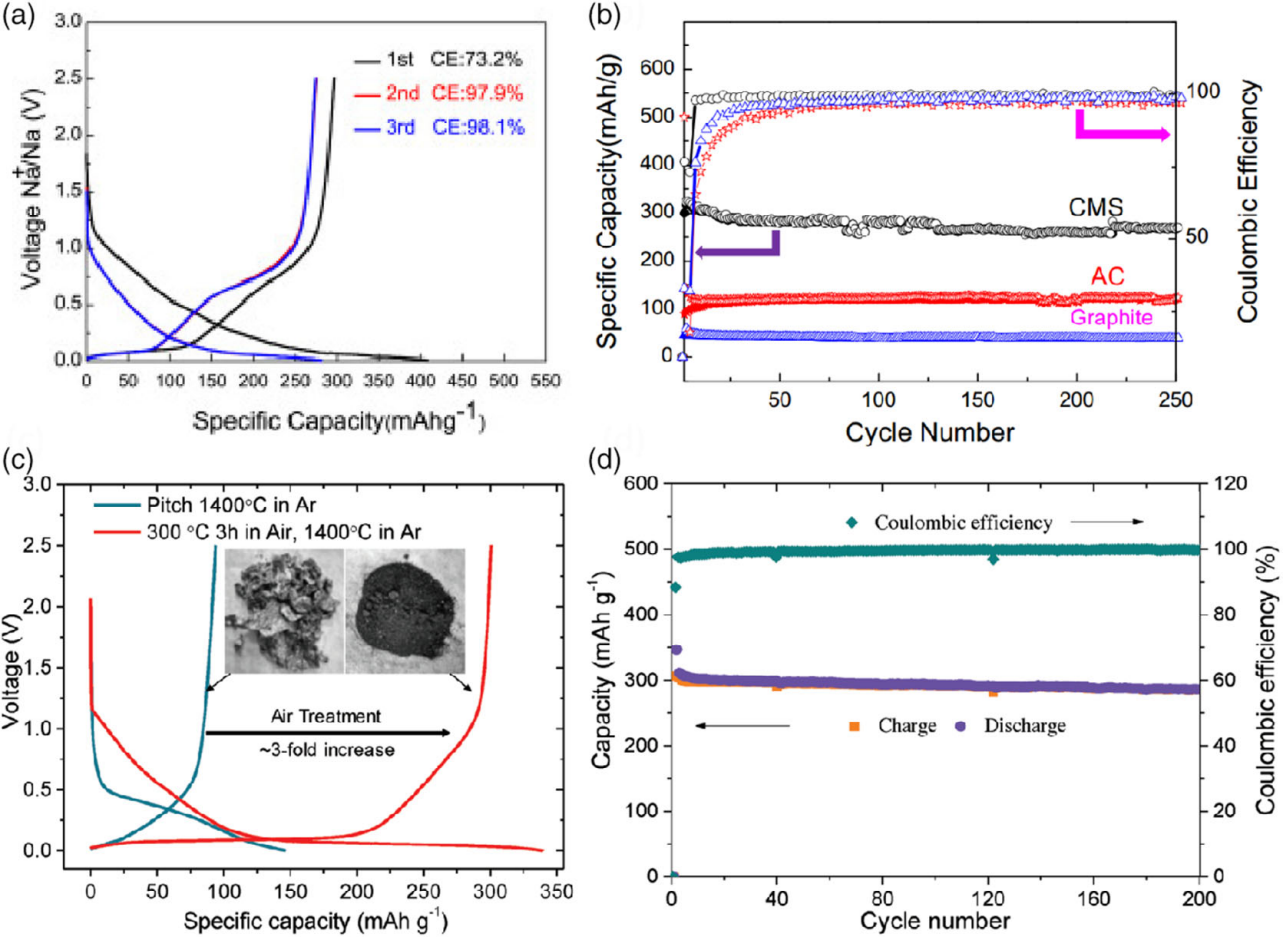
Potential profiles of a) CMS for the initial three cycles. c) CPP‐1400 and CPOP‐1400 for the first cycle. Cycling performance and coulombic efficiency of b) CMS, AC, graphite, and d) CPOP‐1400 at 0.1 C rate. a,b) Reproduced with permission.^[^ ^90^ ^]^ Copyright 2016, Elsevier. c,d) Reproduced with permission.^[^ ^91^ ^]^ Copyright 2018, Wiley‐VCH.

Control of the microstructure is one of the hottest topics for research into hard carbon. Yang et al. proposed a commercial carbon molecular sieve (CMS) as a promising anode which has a large number of ultrasmall micropores (0.3–0.5 nm).^[^ ^90^ ^]^ These pores potentially promote both the desolvation and sodium storage process, thus delivering a high reversible capacity close to 300 mA h g^−1^ and an ICE over 70%, as shown in **Figure** **5**. In addition, Hu et al. proposed a simple strategy of preoxidation in air followed by a thermal reduction at 1400 °C for pitch (CPOP‐1400) to produce an effective structural conversion from ordered to disordered during carbonization.^[^ ^91^ ^]^ Compared with the carbonized original pitch (CPP‐1400), the carbonized preoxidized pitch increases the carbon yield from 54% to 67%, the sodium storage capacity from 94.0 to 300.6 mA h g^−1^, and the ICE from 64.2% to 88.6%, as shown in Figure 5.

**Figure 6:**
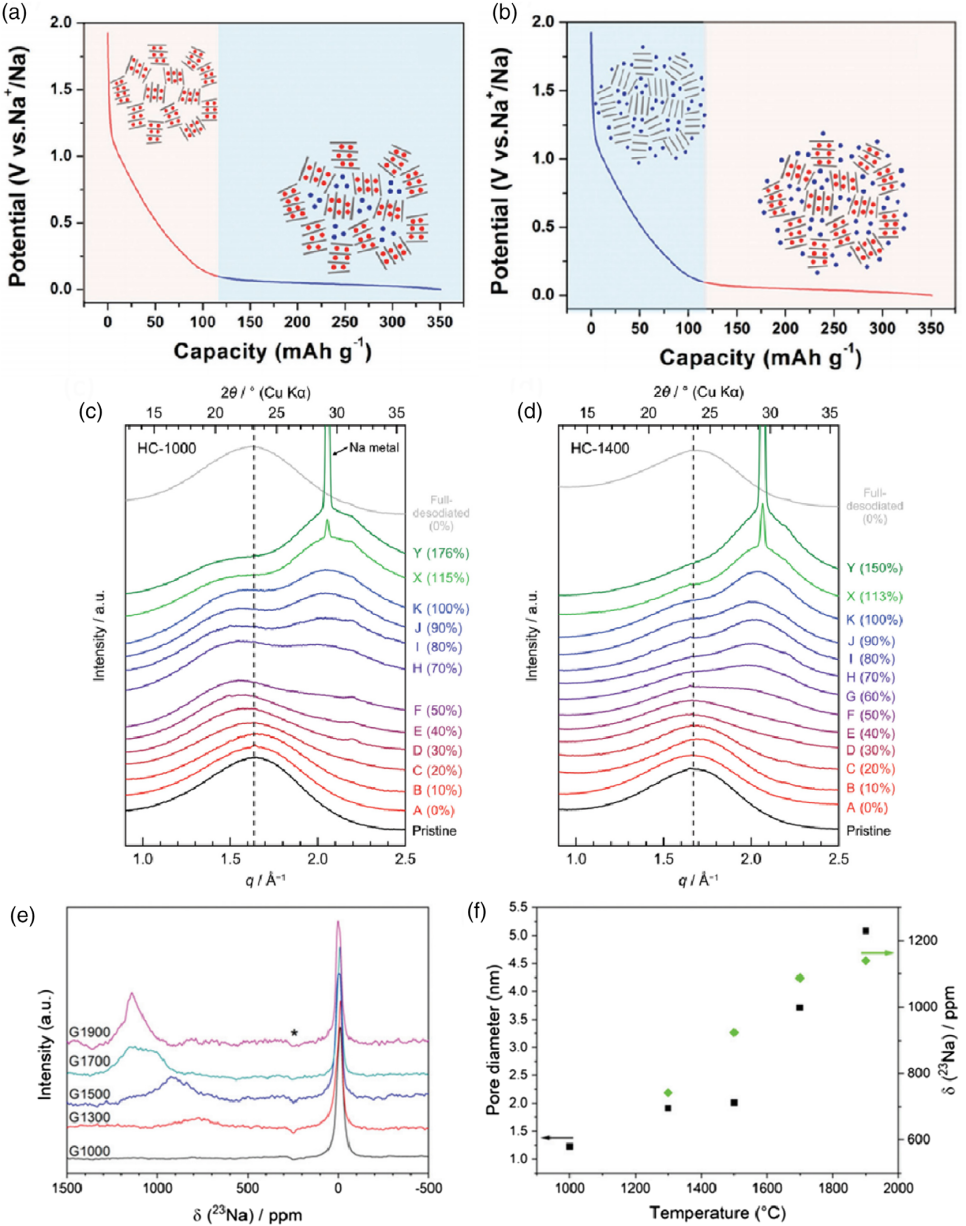
Schematic illustrations of the different charge storage processes related to the low‐voltage plateau: a) sodium storage inside micropores and b) sodium storage between graphene–graphene interlayers. Reproduced with permission.^[^ ^93^ ^]^ Copyright 2017, Wiley‐VCH. Ex situ WAXS patterns of c) HC‐1000 and d) HC‐1400. Reproduced with permission.^[^ ^94^ ^]^ Copyright 2019, Wiley‐VCH. e) Ex situ ^23^Na NMR of G1000–G1900 samples after discharging to 0.005 V. f) Relationship between the pore diameter, quasi‐metallic Na peak shift, and sample pyrolysis temperature. e,f) Reproduced with permission.^[^ ^95^ ^]^ Copyright 2020, The Royal Society of Chemistry.

Understanding the mechanism is the toughest but most crucial topic concerning the use of hard carbon in SIBs. Different from graphite, in which the low‐voltage plateau region is related to the formation of GICs, there is heated debate on what kind of sodium storage process is related to the low‐voltage plateau region. Dahn et al. initially proposed that it was due to sodium storage inside the micropores^[^ ^92^ ^]^ but after many years additional work suggested another possibility, which is that sodium ions intercalate into the graphene–graphene interlayers. Schematics for both mechanisms are shown in **Figure** **6**a,b.^[^ ^93^ ^]^ It was not until recently that more solid evidence was provided and a clearer mechanism proposed. Yamada et al. conducted in situ small and wide angle X‐ray scattering (SAXS/WAXS) on a series of typical sucrose‐derived hard carbons (abbreviated as HC‐1000 and HC‐1400), showing clear evidence of sodium storage inside the closed nanopores of hard carbons.^[^ ^94^ ^]^ Combined with a theoretical simulation, it has been proven that sodium is densely confined inside nanopores to form quasi‐metallic sodium clusters in the low‐voltage plateau, as shown in Figure 6c,d. In addition, Titirici et al. conducted ex situ ^23^Na solid‐state nuclear magnetic resonance (NMR) and neutron total scattering on pyrolyzed glucose (abbreviated as G1000–G1900), which also showed the formation of increasingly metallic sodium clusters on the pore surface of hard carbons in the low‐voltage plateau as shown in Figure 6e,f, and this has been confirmed by density function theory (DFT) calculations.^[^ ^95^ ^]^

Interfacial electrochemistry is another challenging but critical topic for hard carbon anodes. Learning from the industrial experience with graphite in LIBs, the ICE should be higher than 90%, while most hard carbon anodes in SIBs have an ICE around 70–90%. Regulating the electrode structure is the most effective strategy to improve this, including coating it with inert components and reducing the electrolyte‐available carbon surface.^[^ ^96^, ^97^, ^98^, ^99^ ^]^ Another effective strategy is to modify the electrolyte, which directly decides the formation process and specific composition of the SEI. It is interesting to find that ether‐based electrolytes are promising in SIBs.^[^ ^100^, ^101^ ^]^ Indeed, the interfacial electrochemistry of SIBs needs much further research on the processes occurring and this should have a decisive effect on their electrochemical performance.

### 3.3 Carbon Anodes for PIBs

**Figure 7:**
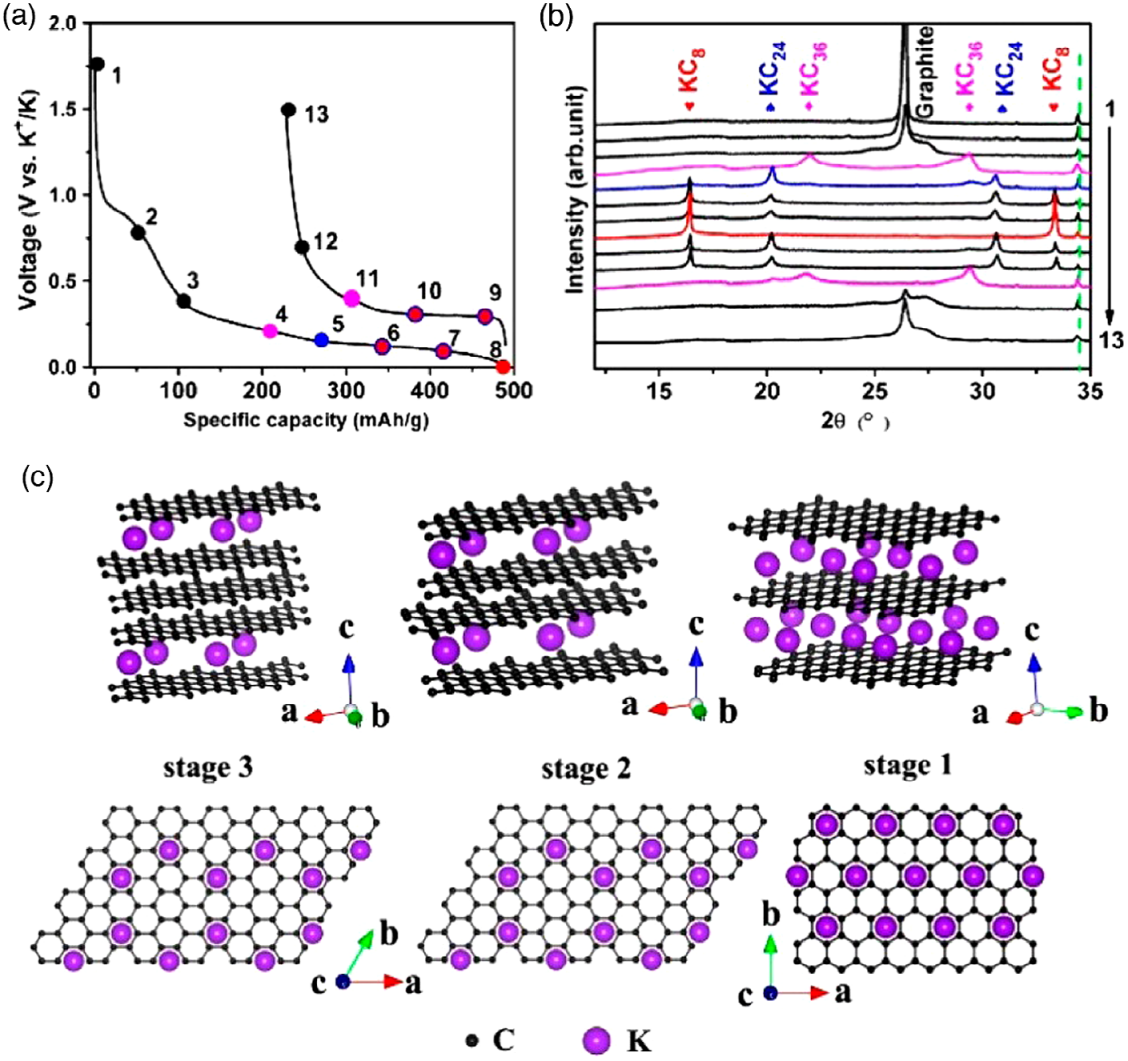
a) Potential profiles of a graphite anode in PIBs. b) Ex situ XRD patterns at selected charge/discharge states. c) Illustrations of the structure of different potassium GICs. a–c) Reproduced with permission.^[^ ^49^ ^]^ Copyright 2015, American Chemical Society.

Similar to sodium, potassium is another abundant alkali metal element, which can be refined with a much lower cost than lithium. It is also worth mentioning that potassium has a lower reduction potential than sodium, allowing PIBs to operate in a wider potential window. In addition, KPF_6_, the commonly used electrolyte salt, is cheaper and safer to synthesize than its lithium or sodium analogues.^[^ ^102^ ^]^ Graphite has been demonstrated to be a decent option for the anodes and the formation of low stage GICs is thermodynamically feasible, similar to LIBs.^[^ ^103^ ^]^ Ji et al. first reported the electrochemical intercalation of K^+^ into graphite, delivering a reversible capacity over 270 mA h g^−1^ in EC‐based electrolytes.^[^ ^49^ ^]^ This value is close to the theoretical capacity of KC_8_. Ex situ X‐ray diffraction (XRD) provided solid proof of the formation of different‐stage GICs during discharge, as shown in **Figure** **7**. However, the specific capacity drops quickly during prolonged cycling. A possible reason for this could be the large volume expansion induced by the intercalation of potassium ions and the unstable SEI produced by EC‐based electrolytes in PIBs.

Nongraphitic carbons have also been explored as alternatives to graphite, which could relieve the severe volume change that occurs during potassium storage.^[^ ^104^, ^105^, ^106^, ^107^, ^108^ ^]^ Similar to its lithium and sodium counterparts, soft carbons also give sloping potential profiles in PIBs and are different from graphite. However, they have been reported to have a superior cycling ability than graphite. Hard carbon has also attracted attention for its potential use in SIBs; however, it gives sloping potential profiles without any plateau region, implying different charge storage mechanisms. As graphite is tested to present inferior rate capability, nongraphitic carbons are attracting increasing attention to be developed as practical anode with high‐power performance in PIBs.

Though the number of studies related to carbon anodes in PIBs is rapidly increasing, the design of carbon anodes with an optimized microstructure is still at an early stage and their ICE and cycling stability need to be improved to meet practical demands. Another critical issue that needs to be addressed is the development of compatible electrolytes to regulate the interfacial electrochemistry of carbon anodes in PIBs.

### 3.4 Summary

Carbon materials are the preferred option for commercial anodes in AMIBs. Graphite is irreplaceable in LIBs and has shown a similar potential in PIBs while additional structural modification and electrolyte optimization are needed. In contrast, hard carbon rather than graphite could deliver the desired electrochemical performance in SIBs as discussed earlier, while the exact charge storage mechanism needs confirmation. In both SIBs and PIBs, we expect carbons to be designed for a practical high‐energy anode in the near future, meeting the ever‐increasing energy storage demands. In addition, specific types of soft and hard carbons will also be developed for use in AMIBs, which could realize fast charging and discharging without a great sacrifice of energy density, and these will have potential use in high‐power applications.

## 4 Designing Efficient Carbon Frameworks for Alloy Anodes

LIBs have already been commercialized for consumer electronics and electric vehicles and are still under intensive study from a scientific perspective to meet the increasing demand for lower cost, higher energy density, and improved safety. As discussed, graphite is now the irreplaceable anode choice for commercial LIBs, which can deliver a high reversible capacity and excellent cycling stability over 500 cycles. However, it has a specific capacity limit of 372 mA h g^−1^, which makes it unable to meet future needs. Alloys are supposed to be the anodes of the future because they can theoretically store many more lithium ions at a relatively low operating potential (below 1.0 V). However, the formation of new phases during alloying leads to an unavoidable volume expansion, which is often higher than 200% and repetitive after the first cycle. Moreover, the fracture or pulverization of alloy anodes is commonly observed and results in an inferior cycling stability, which needs to be well controlled. Though the most promising alloy anodes are different in different AMIBs such as silicon in LIBs and phosphorus in SIBs, their critical problems are similar and thus may be solved by common strategies.

As discussed in Section 2.2, there are many solutions to protect alloy anodes from severe volume expansion and improve their cycling stability. Among them, the design and construction of composite electrodes is the most practical solution.^[^ ^109^, ^110^, ^111^, ^112^, ^113^, ^114^, ^115^, ^116^ ^]^ Due to their simple production methods, environmental friendliness, controllable microstructure and surface chemistry, and excellent electronic conductivity, carbon materials have the greatest potential to combine with alloys. To review and summarize the representative carbon framework designs for alloy anodes in AMIBs, silicon is a specific model and is used as an example here because of its vital importance for both science and industry in LIBs.^[^ ^117^, ^118^ ^]^ Research experience with a silicon anode in LIBs may help guiding future designs for smart carbon frameworks for other alloy anodes, and even extend to SIBs or PIBs.

Carbon materials typically act as conductive additives or frameworks for silicon anodes. Many different carbon materials such as carbon black, graphite, and graphene can be mixed with silicon particles to increase the electronic conductivity and improve the structural stability.^[^ ^115^, ^116^, ^117^, ^118^, ^119^, ^120^ ^]^ Depending on the size and shape of the silicon particles, the addition of a thin, uniform carbon coating is another effective way to alleviate the volume expansion and restrict SEI formation.^[^ ^67^, ^76^ ^]^ Electrostatic interaction and chemical bonding are two common approaches to immobilize silicon nanoparticles on the carbon framework.^[^ ^121^ ^]^ Whereas, as discussed in Section 2.2, to promote the utilization of the electrochemically active silicon particles and maintain their spatial integrity as well as the electrical conductivity of the composite electrode, a 3D carbon framework is widely accepted as the most promising solution. In the following section, the design of the carbon framework is divided into two types, namely, passive and proactive, depending on whether existing commercial carbons is used or synthesizing novel carbons with designed microstructure to combine with silicon anode.

### 4.1 Passive Design of Carbon Frameworks for Alloy Anodes

Carbon materials have a large variety of microstructures, ranging from lamellar graphite, to spherical fullerene and rod‐like carbon nanotubes, and most commercial carbons have 3D interconnected frameworks. Therefore, incorporating silicon into existing carbons is a promising approach to structural design, and is here defined as the passive design of a carbon framework. In this way, a carbon framework with spatial interconnections and structural stability can be used as a high‐capacity alloy anode.

**Figure 8:**
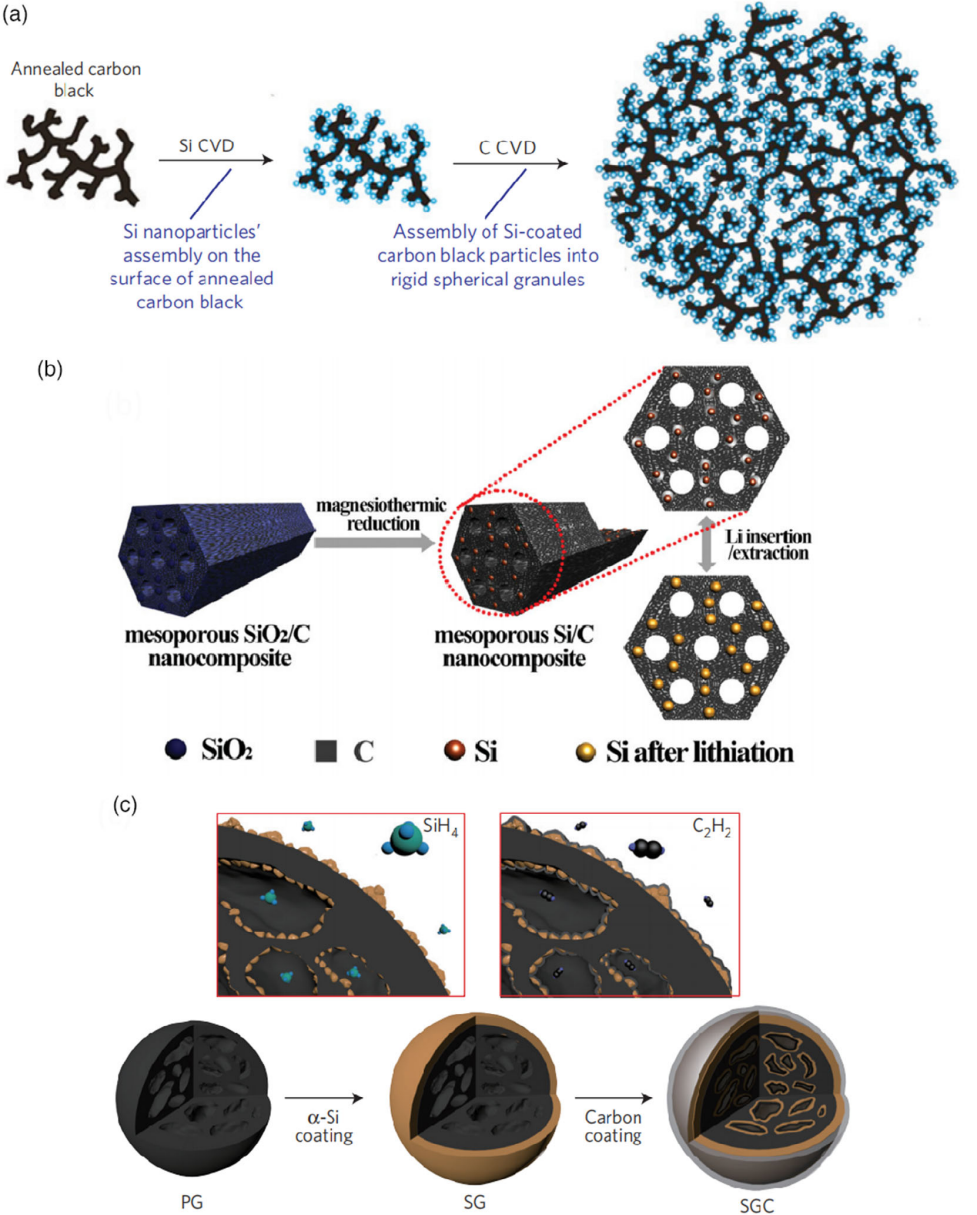
a) Schematic of Si–C nanocomposite formation. Annealed carbon black particles are coated by Si nanoparticles and then assembled into rigid spheres with open interconnected internal channels during C deposition. Reproduced with permission.^[^ ^122^ ^]^ Copyright 2010, Springer Nature. b) A mesoporous Si/C nanocomposite prepared by magnesiothermic reduction. Reproduced with permission.^[^ ^123^ ^]^ Copyright 2014, Wiley‐VCH. c) Schematic of the fabrication process for the SGC hybrid. Gas phase of SiH_4_ and C_2_H_2_ can easily adsorb and penetrate on a pristine graphite (PG) surface and beneath its inner pores, which leads to homogeneous silicon and carbon coating. Reproduced with permission.^[^ ^124^ ^]^ Copyright 2016, Springer Nature.

Yushin et al. select annealed carbon black chains as both the carbon framework and substrate for the chemical vapor deposition (CVD) of silicon nanoparticles,^[^ ^122^ ^]^ as shown in **Figure** **8**a. The robust carbon black spheres with plenty of porosity could accommodate the large volume change of silicon/carbon anode and their irregular channels facilitated the rapid diffusion of lithium ions. Zhao et al. designed a magnesiothermic reduction approach to synthesize mesoporous Si/C nanocomposites, in which ultrasmall and uniform silicon nanoparticles are embedded in a stable mesoporous carbon framework,^[^ ^123^ ^]^ as shown in Figure 8b. The interconnected porosity of the mesoporous carbon framework provided abundant void space to accommodate the large volume expansion of the anode. Cho et al. prepared Si‐nanolayer‐embedded graphite/carbon hybrids (SGC),^[^ ^124^ ^]^ as shown in Figure 8c. This design is scalable and provides outstanding capacity retention even with a high electrode density (>1.6 g cm^−3^).

For commercially available carbons, the already existing carbon frameworks have acceptable spatial interconnection and structural stability, but cannot be adjusted to accept different silicon precursors and microstructures. Special treatment of the silicon precursor is required to adapt it to the specific carbon framework, which leads to a higher cost and complexity of constructing the composite electrode. Moreover, the desired interfacial cohesion between the carbon framework and the silicon is not easily accomplished due to the difficulty of introducing electrostatic interaction or chemical bonding. Therefore, flexible design of the carbon framework is needed, which is defined as proactive design, as discussed later.

### 4.2 Proactive Designs of Carbon Frameworks for Alloy Anodes

Carbon materials can be prepared from different precursors and by different techniques. Their production is simple and their microstructure and surface chemistry can be adjusted by changing the processing parameters. Starting from the mixing of the carbon and silicon precursors to the synthesis of the composite electrode, their interfacial cohesion can be improved by chemical bonding or electrostatic interaction. In addition, for specific silicon precursors, stable and spatially interconnected carbon frameworks can be simply and cheaply customized and synthesized with a low cost, regardless of the physical or chemical properties of the silicon. This proactive carbon design can dramatically increase the reversible capacity, ICE, and prolonged cycling stability of alloy anodes.

Cui et al. designed and fabricated a yolk‐shell structure by sealing commercially available Si nanoparticles inside conformal, thin, and self‐supporting carbon shells.^[^ ^125^ ^]^ The void space between the silicon particles and the carbon shell was designed to allow the silicon to expand freely in the protective carbon framework and also stabilize the SEI on the outside of the carbon shell without continuous formation and break up. Cui et al. also proposed a pomegranate‐like carbon framework,^[^ ^118^ ^]^ where each silicon nanoparticle is wrapped by a conductive carbon layer with reserved void space to form a hybrid nanoparticle which is further encapsulated by a thicker carbon layer to form micron‐size pouches that act as an electrolyte barrier. This carbon framework provided new insight into the tough trade‐off between a high surface area and a low tap density of the framework.

**Figure 9:**
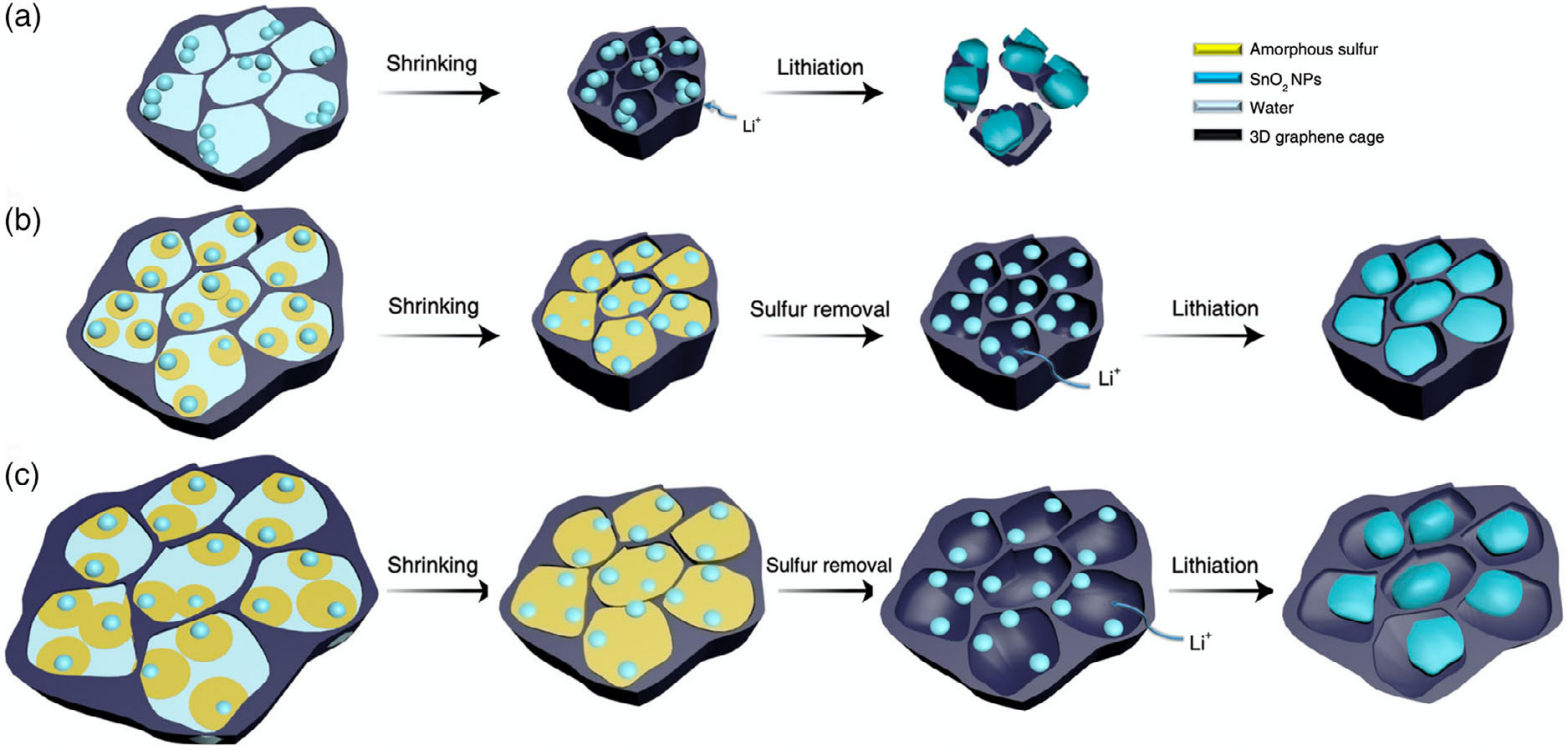
Sulfur template control of the incorporated void space in the graphene framework. a) Schematic of the limited space produced inside the original graphene framework, leading to cracking of the graphene framework and electrode pulverization when the tin oxide expands during the lithium storage process. b) When an appropriate amount of sulfur is introduced into the graphene framework, the tin oxide has enough space for lithiation. As the removal of sulfur results in the smallest but sufficient void space for volume expansion of tin oxide, a largely improved volumetric performance can be achieved. c) The removal of excess sulfur would introduce a large void space and compromise the volumetric performance. a–c) Reproduced under the terms of the CC‐BY 4.0 license.^[^ ^126^ ^]^ Copyright 2018, The Authors, published by Springer Nature.

Recently, Yang et al. reported a 3D graphene framework with a high density but well‐defined void space, and also introduced a sulfur sacrificial template to control the use of the void space.^[^ ^126^ ^]^ The flowable and deformable sulfur, as a soft template, tightly encapsulated the tin oxide as the alloy anode and removal of the sulfur could precisely change the distributed void space to accommodate the volume expansion of the alloy anode, as shown in **Figure** **9**. By adding and caging tin oxide in the flexible and high‐density graphene framework, a large increase in the reversible capacity after hundreds of cycles was obtained and the volumetric performance was also significantly improved. The use of sulfur as a soft template may inspire future proactive carbon designs and may be extended to other novel carbon materials.

Similarly, high‐capacity alloy anodes have attracted much attention in SIBs and PIBs. Therefore, for both passive and proactive carbon designs, carbon materials can play an indispensable role in developing high‐capacity alloy anodes for use in AMIBs. With more insight into the structure regulation and chemistry modification of carbon materials, there may be better proactive carbon framework designs for future commercial applications.

## 5 Summary and Prospects

The increasing need for high‐performance anode materials has provoked research related to carbon materials in AMIBs. In this review, two primary roles of carbon materials have been introduced, as practical anodes and as an efficient framework for high‐capacity alloy anodes. For practical carbon anodes, a clear understanding of the charge storage mechanism, control of the microstructure, and matching electrolytes are critical and thus the optimum carbon anode is different in different batteries. Specifically, graphite is the best option for both LIBs and PIBs, while hard carbon is the best option for SIBs. For an efficient carbon framework for alloy anodes, interfacial cohesion, spatial interconnection, and structural stability are critical. We have highlighted the superiority of proactive carbon designs over passive strategies, due to their excellent compatibility with the alloys and controllable synthesis of a desired framework, which can guarantee cycling stability and improved volumetric performance. Furthermore, the research history and representative studies have been carefully reviewed and discussed, to provide a clear picture of how the right carbon is selected and developed, which could potentially stimulate more research and progress in this field. However, there is still a gap between commercial demand and the present level of research on carbon‐based anode designs for AMIBs: 1) The successful commercialization of the graphite anode has a profound impact on the wide application of LIBs. To some extent, it is the reversible GIC formation and regulated interfacial electrochemistry that makes graphite stand out. Therefore, to develop commercial carbon anodes for both SIBs and PIBs, the charge storage mechanisms have to be precisely characterized, which would provide a guideline for designing low‐cost but high‐performance carbon anodes. For hard carbons in SIBs, their amorphous structure brings complexity on investigating their structural evolution. Characterizing the exact state of sodium at different electrochemical stage should be a better approach to understand the sodium storage mechanism of hard carbons in SIBs. In contrast, for graphite in PIBs, due to its crystal structure, it is better to apply diffraction‐based techniques to track the structural evolution to understand the potassium storage mechanism of graphite in PIBs. In addition, well‐matched electrolytes also need to be developed, with the aim of modifying the electrochemical interface between the carbon anode and the electrolyte and increase the ICE and cycling stability for industrial requirements. To facilitate efficient charge storage, large‐area carbon surface should be reduced and slight coating is also needed to regulate the intense interfacial reaction. 2) Developing practical alloy anodes for LIBs has been widely recognized in industry, and silicon is the material of most interest. Extensive efforts have been made to design and construct carbon frameworks to protect the silicon from pulverization with the loss of electrochemical reactivity. However, how to extend the research from the lab to a commercial factory is extremely challenging because the quantity of raw materials needed is increasing exponentially, which requires lowering the cost of both the raw materials and the synthesis process. For example, the manufacturing cost will be reduced largely if micron‐size silicon can be used instead of nanometer, so the carbon framework for the larger particles is particularly important. Moreover, the uniformity of the composite has to be considered to deliver a decent electrochemical performance with a much higher mass loading than in coin cells.

## Conflict of Interest

The authors declare no conflict of interest.
